# Deciphering Haplotype-level Chromosome Conformation Alteration in Down Syndrome by Haplotype-resolved Multi-omics Analysis

**DOI:** 10.1093/gpbjnl/qzaf054

**Published:** 2025-06-12

**Authors:** Chengchao Wu, Tianshu Zhou, Wenfu Ke, Wei Xiong, Zhihui Zhang, Siheng Zhang, Jinyue Wang, Lulu Deng, Keji Yan, Man Wang, Shenglong He, Qi Gong, Chao Ma, Xiaping Chen, Yan Li, He Long, Chong Guo, Gang Cao, Zhijun Zhang

**Affiliations:** Department of Reproductive Medical Center, Hubei Provincial Clinical Research Center for Umbilical Cord Blood Hematopoietic Stem Cells, Taihe Hospital, Hubei University of Medicine, Shiyan 442000, China; Affiliated Hospital of Guangdong Medical University & Key Laboratory of Zebrafish Model for Development and Disease of Guangdong Medical University, Zhanjiang 524001, China; State Key Laboratory of Agricultural Microbiology, Huazhong Agricultural University, Wuhan 430070, China; College of Veterinary Medicine, Huazhong Agricultural University, Wuhan 430070, China; Department of Reproductive Medical Center, Hubei Provincial Clinical Research Center for Umbilical Cord Blood Hematopoietic Stem Cells, Taihe Hospital, Hubei University of Medicine, Shiyan 442000, China; Department of Reproductive Medical Center, Hubei Provincial Clinical Research Center for Umbilical Cord Blood Hematopoietic Stem Cells, Taihe Hospital, Hubei University of Medicine, Shiyan 442000, China; Department of Reproductive Medical Center, Hubei Provincial Clinical Research Center for Umbilical Cord Blood Hematopoietic Stem Cells, Taihe Hospital, Hubei University of Medicine, Shiyan 442000, China; State Key Laboratory of Agricultural Microbiology, Huazhong Agricultural University, Wuhan 430070, China; College of Veterinary Medicine, Huazhong Agricultural University, Wuhan 430070, China; State Key Laboratory of Agricultural Microbiology, Huazhong Agricultural University, Wuhan 430070, China; College of Veterinary Medicine, Huazhong Agricultural University, Wuhan 430070, China; State Key Laboratory of Agricultural Microbiology, Huazhong Agricultural University, Wuhan 430070, China; College of Veterinary Medicine, Huazhong Agricultural University, Wuhan 430070, China; State Key Laboratory of Agricultural Microbiology, Huazhong Agricultural University, Wuhan 430070, China; College of Veterinary Medicine, Huazhong Agricultural University, Wuhan 430070, China; State Key Laboratory of Agricultural Microbiology, Huazhong Agricultural University, Wuhan 430070, China; College of Veterinary Medicine, Huazhong Agricultural University, Wuhan 430070, China; Department of Outpatient Service Center, Taihe Hospital, Hubei University of Medicine, Shiyan 442000, China; Department of Reproductive Medical Center, Hubei Provincial Clinical Research Center for Umbilical Cord Blood Hematopoietic Stem Cells, Taihe Hospital, Hubei University of Medicine, Shiyan 442000, China; Department of Reproductive Medical Center, Hubei Provincial Clinical Research Center for Umbilical Cord Blood Hematopoietic Stem Cells, Taihe Hospital, Hubei University of Medicine, Shiyan 442000, China; Department of Reproductive Medical Center, Hubei Provincial Clinical Research Center for Umbilical Cord Blood Hematopoietic Stem Cells, Taihe Hospital, Hubei University of Medicine, Shiyan 442000, China; Department of Scientific Research, Taihe Hospital, Hubei University of Medicine, Shiyan 442000, China; Department of Outpatient Service Center, Taihe Hospital, Hubei University of Medicine, Shiyan 442000, China; Department of Clinical Laboratory, Shanghai East Hospital, School of Medicine, Tongji University, Shanghai 200120, China; Department of Gynaecology and Obstetrics, Taihe Hospital, Hubei University of Medicine, Shiyan 442000, China; State Key Laboratory of Agricultural Microbiology, Huazhong Agricultural University, Wuhan 430070, China; College of Veterinary Medicine, Huazhong Agricultural University, Wuhan 430070, China; The Brain Cognition and Brain Disease Institute, Shenzhen Institute of Advanced Technology, Chinese Academy of Sciences, Shenzhen 518055, China; Faculty of Life and Health Sciences, Shenzhen Institute of Advanced Technology, Chinese Academy of Sciences, Shenzhen 518055, China; NMPA Key Laboratory for Research and Evaluation of Viral Vector Technology in Cell and Gene Therapy Medicinal Products, Shenzhen Institute of Advanced Technology, Chinese Academy of Sciences, Shenzhen 518055, China; Department of Reproductive Medical Center, Hubei Provincial Clinical Research Center for Umbilical Cord Blood Hematopoietic Stem Cells, Taihe Hospital, Hubei University of Medicine, Shiyan 442000, China

**Keywords:** Haplotype-resolved multi-omics analysis, Chromosome conformation, Down syndrome, Parental allele-specific, Transcriptional regulation

## Abstract

For chromosome abnormalities (CAs), such as Down syndrome (DS), the influence of genomic variations on chromosome conformation and gene transcription remains elusive. Based on the complete genomic sequences from the parents of a DS trisomy patient, we systematically delineated an atlas of parental-specific, haplotype-resolved single nucleotide polymorphisms (SNPs), copy number variations (CNVs), three-dimensional (3D) genome architecture, and RNA expression profiles in the diencephalon of the DS patient. The integrated haplotype-resolved multi-omics analysis demonstrated that one-dimensional (1D) genomic variations including SNPs and CNVs in the DS patient are highly correlated with the alterations in the 3D genome organization and the subsequent changes in gene transcription. This correlation remains valid at the haplotype level. Moreover, we revealed the 3D genome alteration-associated dysregulation of DS-related genes, which facilitates understanding the pathogenesis of CAs. Together, our study contributes to deciphering the coding from 1D genomic variations to 3D genome architecture and the subsequent gene transcription outcomes in both health and disease.

## Graphical abstract

**Graphical abstract:**
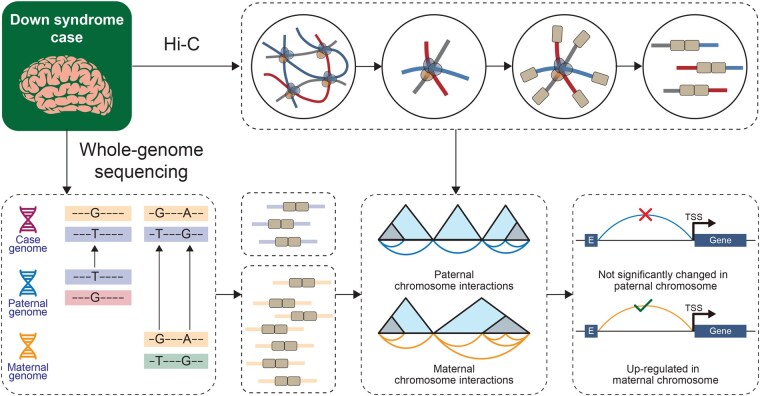

## Introduction

One-dimensional (1D) DNA sequence information of the genome embeds the code of the three-dimensional (3D) chromatin conformation [1]. Variations in the genome, such as copy number variations (CNVs), single nucleotide polymorphisms (SNPs), structural variations (SVs), and chromosome abnormalities (CAs), can influence the 3D chromatin conformation [2–5]. The 3D chromatin conformation can further lead to the alterations of gene transcription and, subsequently, affect the functions and phenotypes [6,7]. Recent advances in high-throughput chromosome conformation capture (Hi-C) technologies have resolved tremendous 3D chromatin conformations of different cells, including spatial elements such as topological associated domains (TADs) and chromatin loops which are highly involved in the regulation of gene expression. However, it remains elusive how the 1D genomic variations influence the 3D chromatin conformation and subsequent gene transcription.

The diploid genome contains two homologous chromosomes of parental origin, with scattered sequence variations within the same nucleus [8]. These variations can be utilized as ideal resources to decipher how the 1D DNA sequence information contributes to the 3D genome architecture. However, most studies in diploid mammals have overlooked the heterogeneity between the two parental chromosomes and captured the average status of 3D chromosome conformation [9–11]. A recent study developed a haplotype-resolved Hi-C analysis pipeline to obtain haplotype-specific information on the 3D genome architecture in mice and its correlation with haplotype-specific gene expression [12]. However, the detailed dogma embedded in the 1D DNA sequence for 3D genome organization and gene transcription regulation is still largely uncharacterized. Moreover, the diploid 3D genome architecture in most organisms remains elusive, which greatly hampers the understanding of the coding of 3D genome structure by 1D DNA sequence.

CA is a serious circumstance of genomic variations, which is caused by the presence of an extra, missing, or irregular portion of homologous chromosomal DNA [13]. Chromosomal aneuploidy can lead to dysregulation of the CA-related gene transcription and subsequent clinical profiles [14]. For instance, Down syndrome (DS) [15], a well-studied CA disease [16], is caused by triplication of human chromosome 21 (HSA21) and characterized by well-defined intelligence disability [17], craniofacial malformation [18], B-cell acute lymphoblastic leukemia [19], early-onset Alzheimer’s disease [20], and other distinctive phenotypic features [21]. Although patients carry an extra HSA21 and thus have 1.5-fold gene dose for genes on this chromosome, the gene expression levels are not uniformly elevated by exactly 1.5 folds, suggesting a sophisticated regulation of the transcription of these genes [22]. However, how this extra chromosome influences overall genome architecture, gene transcription, and subsequent phenotypes of the patients is largely unknown.

Deciphering the haplotype-resolved 3D chromosome conformation in DS can help elucidate the genomic basis and molecular mechanisms underlying the phenotypes associated with CAs. It is also an ideal model to explore the impacts of 1D CAs on haplotype-resolved 3D genome conformation. The aim of this study is therefore to perform an integrated haplotype-resolved multi-omics analysis to deepen the current understanding of the relationship between haplotype-resolved 1D genome sequences and haplotype-resolved 3D genome architecture as well as allele-specific gene regulation at the haplotype level.

## Results

### Integrated haplotype-resolved multi-omics analysis design

**Figure 1:**
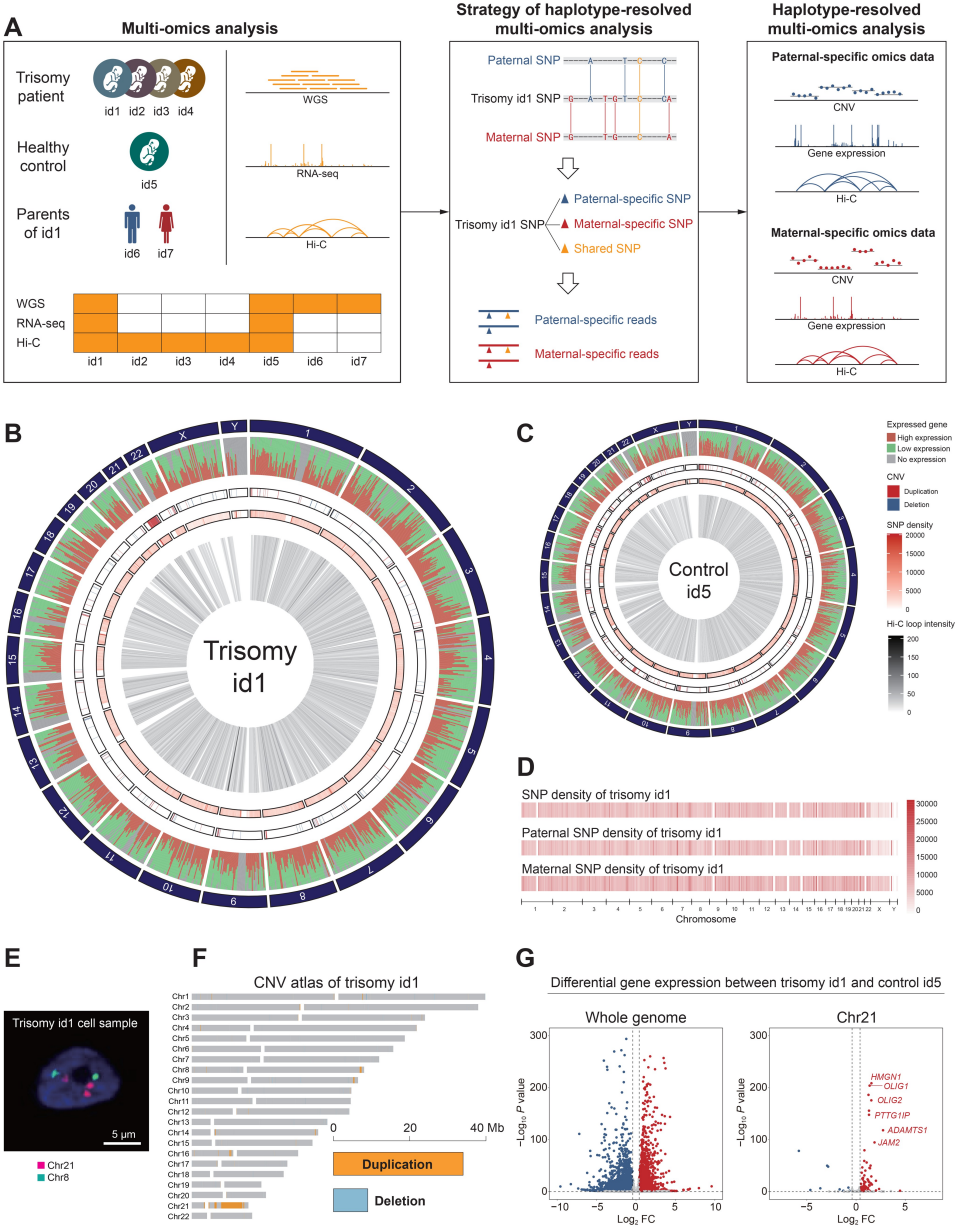
Construction of an integrated haplotype-resolved multi-omics analysis pipeline **A**. Schematic representation of the experimental and analytical design. Blue and red bars represent paternal-origin and maternal-origin unique sequencing fragments, respectively. Similarly, triangles, dots, peaks, and interaction curves in blue and red represent unique SNPs, regions, signal peaks, and interaction intensities in paternal chromosomes and maternal chromosomes, respectively. **B**. and **C**. Circos plots showing multi-omics analyses including gene expression, CNV profiling, SNP density, and Hi-C loop intensity of trisomy id1 (B) and control id5 (C). **D**. SNP densities across the whole genomes of trisomy id1 and the parents. **E**. FISH image of trisomy id1. Burgundy signals represent Chr21 and green signals represent Chr8. **F**. CNV atlas of trisomy id1. Bar plot in the bottom right corner indicates the total length of duplication and deletion in the genome. **G**. Differential gene expression between trisomy id1 and control id5 in whole genome (left) and Chr21 (right). WGS, whole-genome sequencing; CNV, copy number variation; SNP, single nucleotide polymorphism; Hi-C, high-throughput chromosome conformation capture; RNA-seq, RNA sequencing; FISH, fluorescence *in situ* hybridization; Chr, chromosome.

To decipher the 3D chromosome conformation and gene expression alterations in DS, diencephalon samples were obtained from four fetal trisomy patients (named id1–id4) and one healthy control (named id5) (**Figure 1**A). Whole-genome sequencing (WGS), RNA sequencing (RNA-seq), and Hi-C sequencing libraries of these samples were constructed and sequenced (Figure 1A). Then, we performed integrated multi-omics analysis using the CNV, RNA-seq, and Hi-C data (Figure 1B and C). Moreover, to investigate the impact of aneuploidy in trisomy id1 at the haplotype level, we collected blood samples from the parents (id6 and id7) of trisomy id1 and obtained parental WGS data (Figure 1A). With these data, we performed an integrated haplotype-resolved multi-omics analysis to explore the influence of 1D genomic variations on 3D chromosome conformation and allele-specific gene regulation at the haplotype level (Figure 1A).

Based on the WGS data from the parents of trisomy id1, parental-specific SNPs were identified (Figure 1D, Figure S1A and B). Sequencing fragments containing paternal-specific SNPs were named as paternal-specific reads, while those containing maternal-specific SNPs were named as maternal-specific reads (Figure 1A). Using these parental-specific SNPs as markers, all the sequencing libraries were divided into two haplotype-specific datasets for haplotype-resolved CNV, gene expression, and Hi-C analyses to understand the intrinsic relationships between haplotype-resolved genomic variations, haplotype-resolved chromatin architecture, and haplotype-resolved gene transcription.

In the sample of trisomy id1, the presence of an extra HSA21 was confirmed by fluorescence *in situ* hybridization (FISH) (Figure 1E). Our WGS data further demonstrated that the abnormal CNVs mainly occurred on HSA21 (Figure 1F). In contrast, barely any abnormal CNVs were observed on HSA21 in the healthy control (id5) and the parents of trisomy id1 (Figure S1C–E). We performed a comparative analysis of CNVs on HSA21 between the trisomy id1 and control id5, and found that the CNV change ratio in HSA21 was 1.5 (Figure S1F), further validating the presence of an extra HSA21 in the patient. Interestingly, differential gene expression analysis between the trisomy id1 and healthy control id5 (Figure S1G; Table S1) revealed that most differentially expressed genes (DEGs) on HSA21 were DS-related genes (Figure 1G). Furthermore, we analyzed the alternative splicing events on HSA21 and observed alternative splicing events in several DS-related genes in the trisomy id1, such as *HMGN1* and *APP* (Figure S1H).

### Impact of 1D genomic variations on 3D chromosome conformation

**Figure 2:**
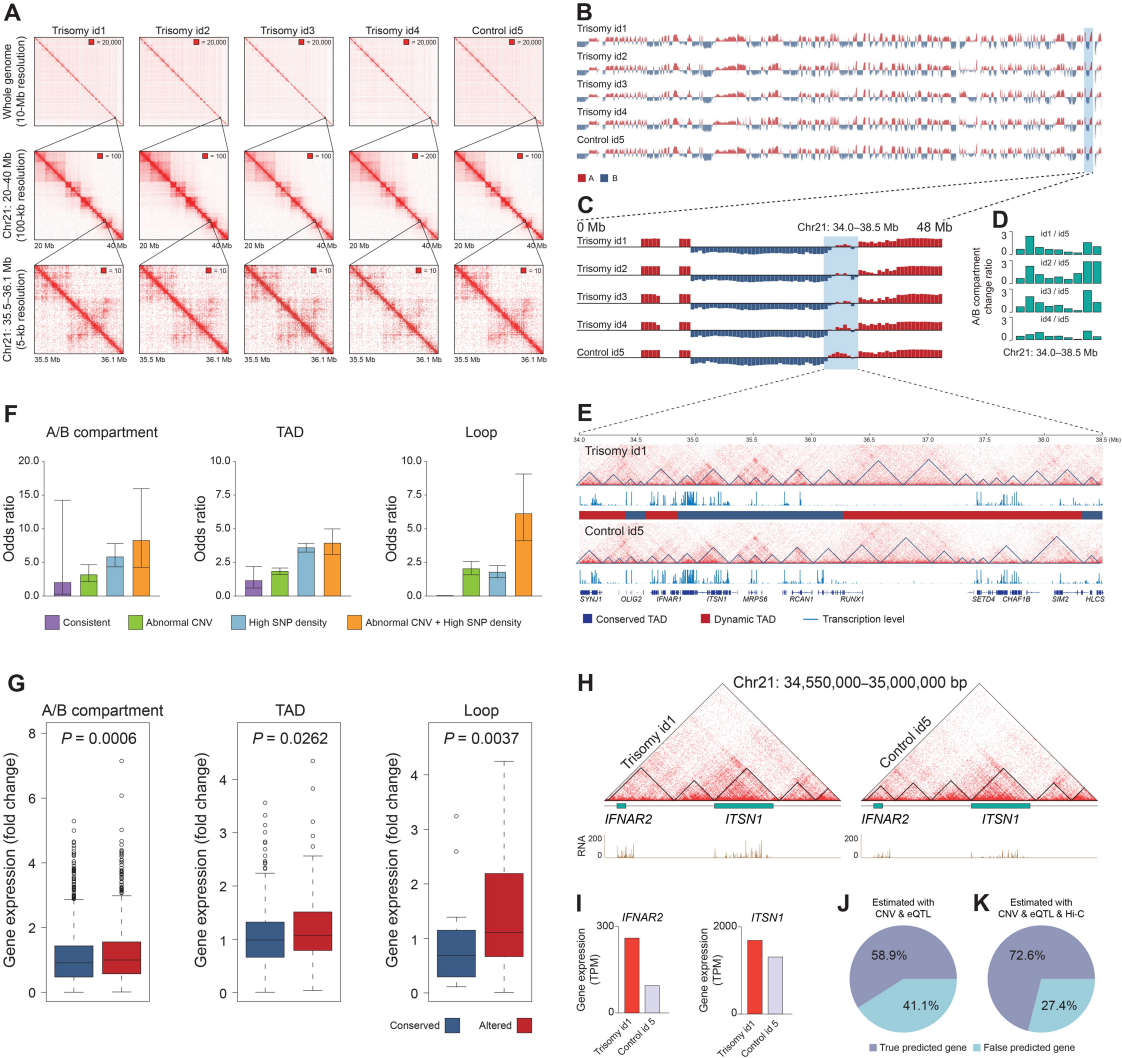
1D genomic variations influence 3D chromosome conformation and gene expression **A**. Spatial chromosome contact maps of trisomy id1–id4 and control id5 at different resolution. Maximum intensity is indicated in the upper right corner of each contact map. **B**. and **C**. Distribution of A/B compartments of trisomy id1–id4 and control id5 across the whole genome (B) and on Chr21 (C). **D**. A/B compartment change ratio in Chr21: 34.0–38.5 Mb. X-axis indicates serial chromosomal regions. See Materials and methods for more details. **E**. Differences of TADs between trisomy id1 and control id5 in Chr21: 34.0–38.5 Mb. **F**. Odds ratio of A/B compartments, TADs, and chromatin loops enriched in different 1D genomic regions. **G**. Box plots showing the fold changes in gene expression between conserved and altered A/B compartments, TADs, and chromatin loops. **H**. TAD altered events within Chr21: 34,550,000–35,000,000 bp and corresponding RNA peaks. **I**. Gene expression of two genes in trisomy id1 and control id5. **J**. Pie chart showing the percentage of true predicted genes using 1D genomic features. **K**. Pie chart showing the percentage of true predicted genes using 1D genomic features and 3D spatial conformation information. TAD, topological associated domain; TPM, transcripts per million; eQTL, expression quantitative trait locus; 1D, one-dimensional; 3D, three-dimensional.

To further understand the impact of trisomy on the spatial chromatin organization and subsequent gene expression, digestion-ligation-only Hi-C (DLO Hi-C) was performed to capture genome-wide chromosome conformation in trisomy id1–id4 and control id5 (**Figure 2**A, Figure S2A; Table S2). While the overall chromatin interaction frequencies in different genomic distances appeared similar between trisomy id1–id4 and control id5, correlation analysis of the contact matrices revealed certain differences between trisomy id1–id4 and control id5 (Figure S2B). We then analyzed the A/B compartments in each chromosome (Figure 2B) and found significant A/B compartment switches in HSA21 (Figure 2C), especially within the region of HSA21: 34,000,000–38,500,000 bp (Figure 2D), which corresponds to 21q22.1, a DS critical region. TADs and corresponding gene expression in this region also showed some alterations (Figure 2E). Moreover, the differences in interchromatin interaction between trisomy id1–id4 and control id5 were investigated (Figure S2C). Notably, the percentage of interchromatin interactions involving HSA21 in trisomy id1–id4 was more than twice that in control id5 (Figure S2D), as illustrated by the differential interchromatin interactions between HSA21 and Chr22 (Figure S2C). These results suggest large-scale alterations in 3D chromatin conformation in these regions.

TADs, loops, and A/B compartments are key elements of genomic spatial architecture (Table S3). Next, we performed an integrated analysis using Hi-C, CNV, and SNP data to investigate the correlation between 1D genomic variations and alterations in 3D conformation. As shown in Figure 2F, altered TADs, loops, and A/B compartments in trisomy id1 were significantly enriched in the regions with abnormal CNVs and high SNP density, suggesting that CNVs and SNPs may influence alterations in spatial conformation. Of note, odds ratio of enrichment analysis demonstrated that the regions with both abnormal CNVs and high SNP density contain significantly more genomic spatial conformation alterations (Figure 2F). These results suggest that 1D genomic variations may influence the 3D conformation of the genome.

### Impact of spatial chromatin organization on gene expression

To investigate the impact of spatial chromatin organization on gene expression, we compared the genomic structure and gene expression between the patient and the healthy control and then performed statistical analysis to evaluate the DEGs between genomic regions with conserved and altered spatial conformation. Notably, genes in regions with altered spatial conformation showed greater expression changes compared to those in regions with conserved spatial conformation, suggesting that spatial chromatin organization may influence gene expression (Figure 2G). For example, two TADs in the region of Chr21: 34,550,000–35,000,000 bp were fused into a single TAD in trisomy id1 (Figure 2H), correlated with a 2.73-fold up-regulation of *IFNAR2* (Figure 2I). In contrast, the expression of *ITSN1*, located in a conserved TAD, increased only 1.31 folds (Figure 2I), a change likely attributable to the abnormal dosage of copy number in HSA21 (expected value: 1.5 folds).

In the Hi-C contact maps of trisomy id1–id4, two obvious loops were observed in the upstream of *RCAN1*, while no significant interaction was detected in the same region of control id5 (Figure S2E). *RCAN1* is a DS-related gene located on HSA21 contributing to long-term potentiation and memory. The H3K27me3 histone modification signal and DNA methylation data from a fetal brain tissue database suggest that the regulatory region in the upstream of this gene most likely functions as a repressor (Figure S2F). Thus, we speculate that a long-range repressive regulation occurs in this region in trisomy id1–id4, which leads to the repression of *RCAN1* expression (Figure S2G), further implying that alterations in spatial chromatin organization can influence gene expression.

### Predicting gene expression regulation in trisomy using 1D genomic features

Inspired by the intrinsic correlation among 1D genomic variations, 3D spatial conformation, and gene expression, we attempted to predict gene expression in trisomy id1 by 1D genomic features (Figure S2H). Generally, gene expression is influenced directly by its copy number. Besides, adjacent allelic SNPs which may alter the binding affinity to the transcription complex also have impact on the expression levels of the gene. Thus, we obtained the copy number of each gene from CNV results, as well as the expression fold change of each gene between allelic genotypes from the Genotype-Tissue Expression (GTEx) portal (https://www.gtexportal.org/). To predict gene expression levels in the healthy control id5 compared to the trisomy patient id1, the putative gene expression levels of the healthy case were corrected with copy numbers and expression quantitative trait locus (eQTL) fold changes. By this approach, the expression levels of 58.9% (2950/5002) of genes were accurately predicted (Figure 2J; Table S4).

To further improve the prediction of gene expression regulation, we incorporated more gene regulatory factors. Our data indicate that spatial conformation is also an important factor in regulatory transcription events. Consistent with this, we observed that the similarity of Hi-C contact matrices in the upstream regions of genes between trisomy id1 and control id5 was significantly higher in the true predicted gene sets than in the false predicted gene sets (Figure S2I; Table S4), suggesting that genomic spatial conformation may contribute to gene expression prediction. Thus, we included spatial conformation features in our gene expression prediction model (see Materials and methods for details). As shown in Figure 2K, the inclusion of spatial conformation features improved the prediction accuracy to 72.6% (3634/5002). This analysis indicates that 1D genomic features and 3D spatial conformation information can be integrated to assess gene expression in a quantitative approach.

### Impact of parental-specific SNPs and CNVs on haplotype-resolved chromatin architecture

**Figure 3:**
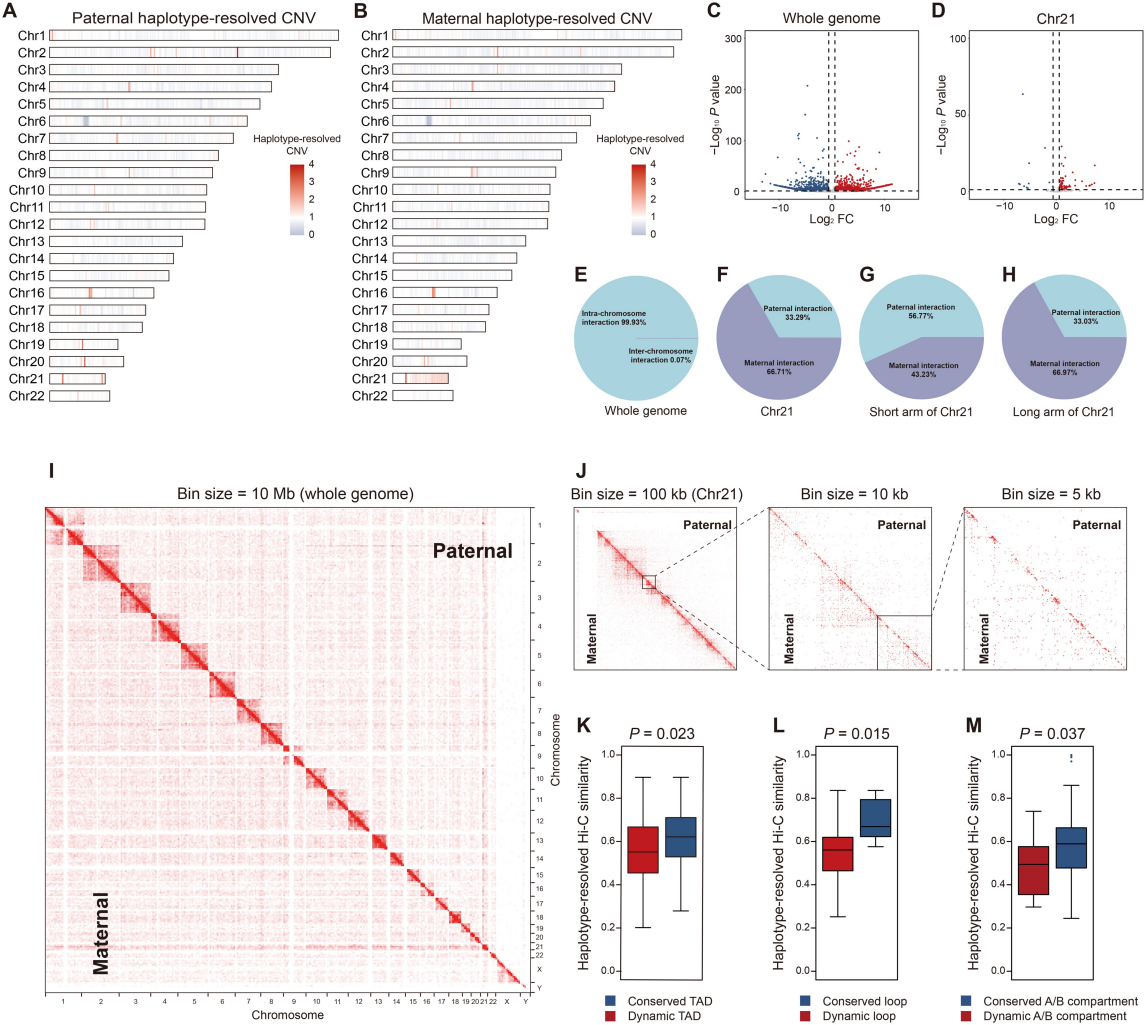
Differences between chromosomes of paternal and maternal origin revealed by haplotype-resolved multi-omics analysis **A**. and **B**. CNV profiles of chromosomes of paternal (A) and maternal (B) origin. **C**. and **D**. Differential gene expression between chromosomes of paternal and maternal origin across the whole genome (C) and in Chr21 (D). **E**. Proportion of intra-chromosome and inter-chromosome interactions across the whole genome. **F**. Proportion of paternal and maternal chromosome interactions in Chr21. **G**. and **H**. Proportion of paternal and maternal chromosome interactions in the short arm (G) and long arm (H) of Chr21. **I**. Heatmap of haplotype-resolved spatial chromatin organization across the whole genome. **J**. Heatmap of haplotype-resolved spatial chromatin organization of Chr21 at different bin sizes. **K**.–**M**. Parental-specific interaction similarity of conserved and dynamic TADs (K), chromatin loops (L), and A/B compartments (M).

As the genome contains two parental sets of homologous chromosomes in the same nucleus, the haplotype-resolved multi-omics analysis might be an ideal approach to explore how the 1D genomic variations shape the 3D genomic structure and gene transcription. To further investigate the subtle differences between the parents at the haplotype level, we performed an integrated haplotype-resolved multi-omics analysis based on the genome sequences from the parents, as described in Figure 1A. First, paternal and maternal haplotype-resolved CNVs were assembled (**Figure 3**A and B). The haplotype-resolved CNV analysis revealed that the abnormal chromosome is maternal origin (Figure S3A–C), and trisomy occurs most frequently in the long arm of HSA21 (Figure S3D). Next, haplotype-resolved gene expression levels were analyzed as illustrated in Figure 3C. Overall, the maternal chromosome showed higher gene expression compared to the paternal chromosome on HSA21(Figure 3D).

To explore the haplotype-resolved 3D genomic architecture, haplotype-resolved Hi-C analysis was performed (Figure 3E–J, Figure S3E). Our data showed that about 99.93% of interaction events occur within chromosomes (Figure 3E, Figure S3F). The number of maternal chromosome interactions on HSA21 is more than twice that of paternal chromosome interactions (Figure 3F), consistent with the duplication observed in the long arm of HSA21 (Figure 3G and H). Using the high-resolution haplotype-resolved interaction heatmaps, we captured the subtle differences in 3D chromatin structure between chromosomes (Figure 3I and J, Figure S3G). The conserved and dynamic haplotype-level chromatin structures of the spatial structure elements (TADs, loops, and A/B compartments) between trisomy id1 and control id5 were analyzed. Notably, compared to conserved TADs between trisomy id1 and control id5, dynamic TADs showed significantly lower haplotype-resolved Hi-C similarity (Figure 3K). Similar trends were also observed between conserved and dynamic loops and A/B compartments (Figure 3L and M), suggesting that genomic regions exhibiting haplotype-resolved Hi-C differences are more prone to undergoing chromatin structure alterations.

**Figure 4:**
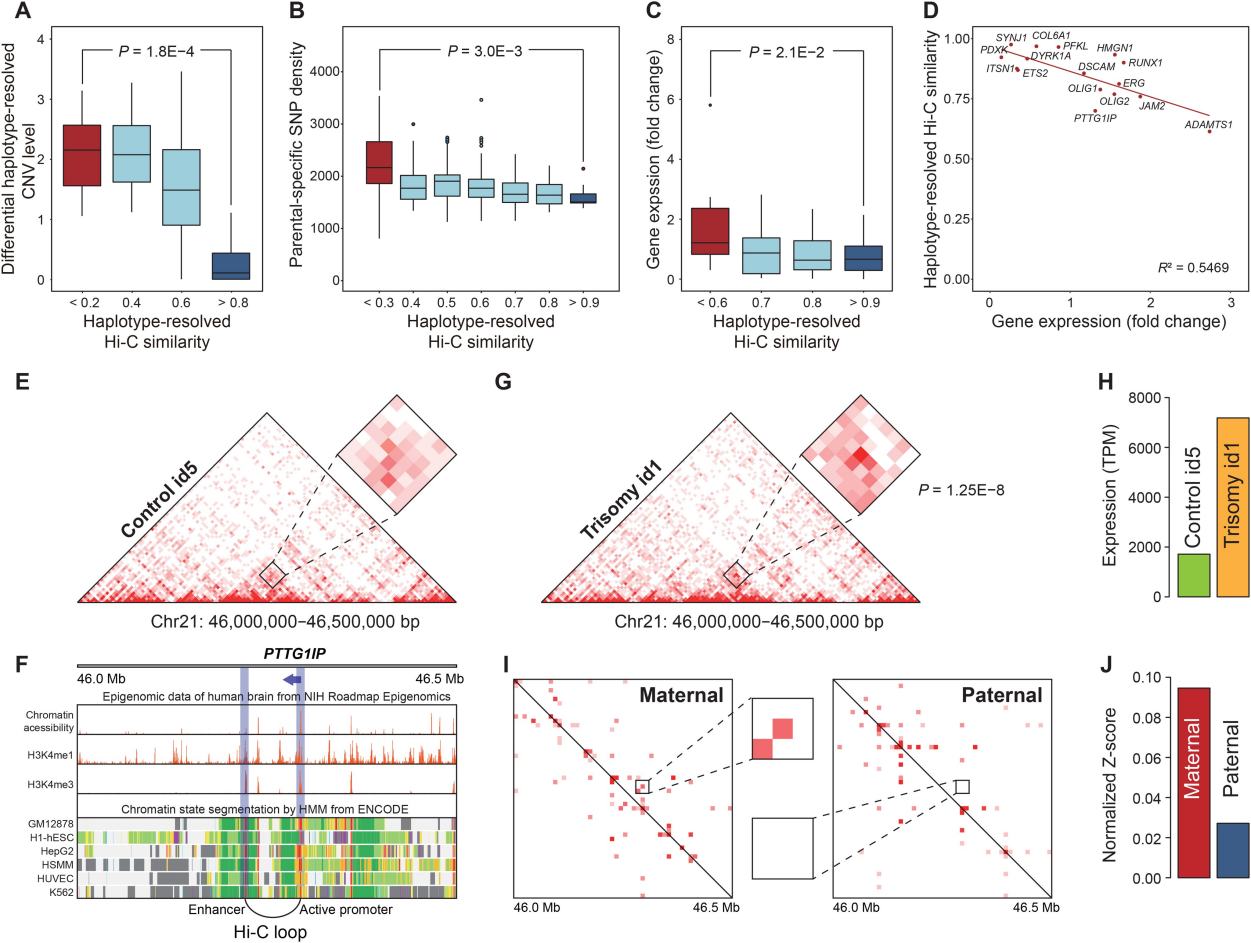
1D genomic variations influence 3D chromosome conformation and gene expression at the haplotype level **A**. Box plot showing differential haplotype-resolved CNV levels at each haplotype-resolved Hi-C similarity level. **B**. Box plot showing parental-specific SNP density at each haplotype-resolved Hi-C similarity level. **C**. Box plot showing fold change of gene expression at each haplotype-resolved Hi-C similarity level. In (A–C), red box represents low haplotype-resolved Hi-C similarity regions and blue box represents high haplotype-resolved Hi-C similarity regions. **D**. Linear correlation between haplotype-resolved Hi-C similarity and differential gene expression for 16 DS-related genes. **E**. Heatmap of Hi-C interactions in Chr21: 46,000,000–46,500,000 bp in healthy control id5. **F**. Chromatin accessibility, histone modifications, and chromatin state segmentation in the promoter and enhancer regions of *PTTG1IP*. **G**. Heatmap of Hi-C interactions in Chr21: 46,000,000–46,500,000 bp in trisomy id1. The loop with a significant *P* value is marked by a rectangle. **H**. Expression of *PTTG1IP* in trisomy id1 and control id5. **I**. Heatmap of parental-specific haplotype-resolved Hi-C interactions in Chr21: 46.0–46.5 Mb. **J**. Standard haplotype-resolved expression of *PTTG1IP* in parental-specific chromosomes. DS, Down syndrome; NIH, National Institutes of Health; HMM, Hidden Markov Model; ENCODE, Encyclopedia of DNA Elements.

Next, we explored the impact of haplotype-resolved CNVs and parental-specific SNP density on haplotype-specific chromatin architecture. As shown in Figure S4A, a strong linear correlation was observed between haplotype-resolved Hi-C similarity and haplotype-resolved CNV similarity. The regions with high haplotype-resolved CNV similarities show high haplotype-resolved Hi-C similarities, and *vice versa* (**Figure 4**A). Of note, conventional Hi-C analysis without haplotype-resolved information did not reveal this correlation (Figure S4B), further supporting the sensitivity and robustness of haplotype-resolved Hi-C analysis.

Furthermore, we calculated the paternal-specific and maternal-specific SNP density (Figure S4C) and analyzed the correlation between haplotype-resolved Hi-C similarity and the haplotype-specific SNP density. As shown in Figure 4B, conserved regions with high haplotype-resolved Hi-C similarity displayed low SNP density heterogeneity, whereas altered regions with low haplotype-resolved Hi-C similarity displayed high SNP density heterogeneity. These results imply that parental-specific SNPs may contribute to shaping haplotype-resolved 3D chromatin architecture. Moreover, transcription factor binding site (TFBS) enrichment analysis was performed in both conserved and altered regions. The results showed that altered regions contained more TFBSs associated with DS-related genes compared to conserved regions, suggesting that these altered regions might make more contribution to the onset of DS (Figure S4D and E).

### Impact of haplotype-resolved chromatin architecture on allele-specific gene expression

To investigate the relationship between haplotype-resolved Hi-C similarity and gene expression, we analyzed their correlation. The results showed that, compared to genes located in regions with high haplotype-resolved Hi-C similarity, those in regions with low haplotype-resolved Hi-C similarity exhibited significantly greater changes in gene expression (Figure 4C). We selected 16 DS-related genes on HSA21 and analyzed the linear correlation between their expression levels and haplotype-resolved Hi-C similarity (Figure 4D). Our results showed that haplotype-resolved Hi-C similarity was strongly associated with the differential expression of these genes, suggesting that haplotype-resolved chromosome conformation may be involved in the regulation of gene expression. This analysis also supports the sensitivity and robustness of haplotype-resolved Hi-C analysis.

Moreover, we analyzed the association between haplotype-resolved Hi-C interactions and haplotype-resolved gene expression of four DS-related genes, and revealed that the haplotype-level chromatin structure can influence allele-specific differential gene expression (Figure S4F–I). Based on the integrated haplotype-resolved multi-omics analysis, we identified an enhancer-mediated regulatory event targeting the *PTTG1IP* gene. As shown in Figure 4E and F, the *PTTG1IP* gene in control id5 exhibited only a weak interaction between the enhancer and promoter. In contrast, in trisomy id1, the interaction between enhancer and promoter was significantly stronger and formed a long-range loop (*P* = 1.25E−8) (Figure 4G), which may lead to a significant increase in gene expression (Figure 4H). Comparison of the same region in the haplotype-resolved Hi-C matrices revealed higher interaction intensity in the maternal chromosome than in the paternal chromosome (Figure 4I). Consistent with this, haplotype-resolved gene expression analysis showed higher expression of the maternal *PTTG1IP* allele than the paternal allele (Figure 4J), implying that stronger enhancer–promoter interactions may specifically facilitate gene expression in a haplotype-specific manner.

## Discussion

It has been reported that genomic variations such as SNPs, SVs, and CNVs embedded in the 1D DNA sequence can shape 3D genome architecture, which subsequently regulates gene transcription. In line with this, our Hi-C analysis revealed that the 3D genome structures, such as TADs, loops, and A/B compartments, indeed underwent some changes in the trisomy cases. Of note, these altered structures were mainly enriched in regions with abnormal CNVs and high SNP density and were correlated with the differential expression of corresponding genes. Importantly, we attempted to predict gene expression in the trisomy cases by combining the 3D genome structures with gene copy numbers and eQTL analysis, which significantly improved the prediction accuracy compared to the approach without genomic structure information. These findings further prove that 1D genomic variations can indeed shape 3D genome architecture and regulate subsequent gene transcription.

As the diploid genome contains two parental-origin homologous chromosomes within the same regulatory environment in the nucleus, genomic variations, especially in CA patients, can be utilized as an ideal model to decipher how 1D DNA sequences encode the 3D genome structure. Compared to the standard Hi-C analysis, haplotype-resolved Hi-C analysis can capture subtle interaction differences between haplotypes and delineate the architecture of each chromosome specifically. Our data demonstrate that haplotype-resolved Hi-C analysis is indeed more sensitive than conventional Hi-C analysis. For example, we observed that the regions with high haplotype-resolved CNV similarities also showed high haplotype-resolved Hi-C similarities, a correlation that was not detected by the conventional Hi-C analysis lacking haplotype-level information. Moreover, we revealed that both haplotype-resolved CNVs and parental-specific SNP density contribute to haplotype-resolved chromosome spatial structure, and that haplotype-resolved Hi-C similarity is strongly associated with the differential expression of corresponding genes. Together, our findings provide haplotype-level evidence that 1D genomic sequences encode 3D chromatin conformation and regulate allele-specific gene expression.

While it is known that the extra chromosome in CA patients can lead to more complex transcriptional regulation of CA-related genes on top of gene dosage effect, it remains elusive how the extra chromosome influences genome architecture, gene transcription, and subsequent phenotypes of the patients. Our findings revealed that the fusion of two TADs in Chr21: 34,550,000–35,000,000 bp in trisomy is correlated with the altered expression of many DS-related genes in this region. For example, *IFNAR2* showed a 2.73-fold up-regulation, significantly higher than the expected1.5-fold dosage effect. Moreover, we observed a significant increase in long-range interactions between the promoter of *PTTG1IP* and an upstream enhancer element in the trisomy case. Further haplotype-resolved Hi-C and RNA-seq analyses indicated that the strong enhancer–promoter interaction in the maternal chromosome may lead to the overexpression of the maternal *PTTG1IP* allele. Together, these results suggest that the extra chromosome in DS patients can alter genome architecture, which may lead to dysregulation of DS-related gene transcription and contribute to the associated phenotypic outcomes.

In summary, our integrated haplotype-resolved multi-omics analysis systematically illustrates the influence of 1D genomic variations on 3D chromosome conformation and gene transcription at the haplotype level. Using the whole-genome sequences from the parents of the DS patient, we delineated an atlas of parental-specific SNPs, CNVs, 3D genome structure, and allele-specific gene expression profiles in the diencephalon of DS patients. Furthermore, we revealed the dysregulation of DS-related genes associated with alterations in 3D genome organization. These findings contribute to understanding the complex transcriptional regulation in DS patients and shed light on the molecular pathogenesis of CAs. It is worth mentioning that genetic information of organisms affects phenotype and disease not only through 3D chromosome conformation but also through other factors, such as epigenetic modifications, transcription factor binding, and chromatin accessibility, as well as post-transcriptional and translational regulation. Therefore, we cannot conclude that this is the only way. On the contrary, we are simply discussing that the regulation of gene expression through 1D genomic information within the context of 3D chromosome conformation is indeed present and logical.

## Materials and methods

### Trisomy and control samples

The five fetal samples (trisomy id1–id4 and control id5) were aborted within 22 weeks, 23 weeks, 20 weeks, 20 weeks, and 26 weeks, respectively. The diencephalon samples of fetal brain were used for constructing omics libraries.

### Library construction and WGS

WGS was performed as follows: 1 ml of a well-mixed blood sample was added into a 15-ml RNase-free centrifugal tube containing 3 ml of erythrocyte lysis buffer and mixed by inversion. After repeated inversion for 10 min, the sample was centrifuged at 2000 r/min for 3 min, and the precipitate was collected. The leukocyte pellet was loosened and resuspended by vortexing. Genomic DNA was extracted using TIANamp Genomic DNA Kit (Catalog No. DP304-02, TIANGEN Biotech, Beijing, China), fragmented by ultrasonication, and subjected to end repair and A-tailing. Finally, library amplification was performed using Illumina sequencing adaptors, followed by sequencing.

### Library construction and RNA-seq

Total RNA was extracted from the diencephalon tissue using TRIzol Reagent (Catalog No. 15596026, Invitrogen, Carlsbad, CA). Then, RNA-seq library was constructed using the VAHTS Stranded mRNA-seq Library Preparation Kit for Illumina (Catalog No. NR602-01, Vazyme, Nanjing, China) following the manufacturer’s protocol.

### DLO Hi-C experiment

The DLO Hi-C experiment was performed as previously described [23].

### SNP calling

Sequencing reads were processed using Trim Galore (v0.6.6, https://github.com/FelixKrueger/TrimGalore) for quality and adapter trimming. The filtered reads were then aligned to the *Homo sapiens* reference genome (hg19) using Burrows-Wheeler-Alignment (v0.7.17) [24]. Subsequently, SNP calling was performed following the Genome Analysis Toolkit (v4.0.12.0) [25] best-practice guidelines, including removal of duplicate reads, recalibration of base quality scores, and variant calling for each sample. The resulting SNPs were then subjected to variant quality score recalibration (VQSR) with a batch sensitivity threshold of 99% to eliminate false positives. Output files were generated in the common variant call format (VCF).

### CNV calling

We used CNVnator (v0.4.1) [26] to detect CNVs from the WGS data of each sample, with different window sizes (trisomy id1: 1500 bp, control id5: 1900 bp, pater: 2700 bp, mater: 3200 bp) to fit the mean property to sigma value. For the CNVnator outputs, we removed calls with q0 > 0.5 or q0 = −1, eval1 ≥ 0.05, and eval2 ≥ 0.05 within CNV regions.

### Gene expression analysis

FastQC (v0.11.8) was employed to assess the quality of the RNA-seq reads, and Trimmomatic (v0.33) [27] was used to filter out low-quality bases and adapter sequences. HISAT2 (v2.1.0) [28] was utilized to align the cleaned reads to the *Homo sapiens* reference genome (hg19) supplemented with Ensembl transcriptome annotations. Read counts for each gene were quantified from the aligned reads using HTSeq (v0.13.5) [29]. Differential expression analysis was performed using DESeq2 (v1.36.0) [30], including normalization of read counts and identification of DEGs.

### Alternative splicing analysis

Alternative splicing events were quantified using SUPPA2 (v2.3) [31]. Differences in present spliced-in (ΔPSI) of alternative splicing transcripts were calculated and used as the standard metric for quantifying splicing variations between samples.

### DLO Hi-C analysis

DLO Hi-C tool (v0.3.9) [32], an integrated computational pipeline for DLO Hi-C analysis, was used to process the Hi-C data and generate contact matrices. The Hi-C interaction libraries were randomly downsampled to the same size with the seqtk toolkit for further differential analysis.

### Hi-C matrix similarity

Hi-C matrix similarity was assessed by calculating the Pearson correlation coefficient between Hi-C matrices. Hi-C matrices at different resolutions were generated using HiCExplorer (v3.6) [33].

### Hi-C loop calling

The hicDetectLoops tool within HiCExplorer (v3.6) [33] was used to detect enriched interaction loops. For altered chromatin loop definition, a pair of loop anchors from two compared matrices was classified as conserved if the differential distance between their anchor positions was within 5 windows. Otherwise, the loops were considered altered.

### TAD boundary calling

The hicFindTADs tool within HiCExplorer (v3.6) [33] was used to identify TAD boundaries. For altered TAD definition, two TADs from two compared matrices were classified as conserved if their overlapping region was higher than 90% of the larger TAD region. Otherwise, the TADs were considered altered.

### A/B compartments

The hicPCA tool within HiCExplorer (v3.6) [33] was used to perform principal component analysis (PCA) on the Hi-C contact matrices with 1-Mb bin size. The first eigenvector [principal component 1 (PC1)] was used to assign A and B compartments. Bins with PC1 > 0 were considered to be A compartments, and bins with PC1 < 0 were considered to be B compartments. The A/B compartment change ratio was calculated as follows:


(1) $$\mathrm{Chang}e\;\mathrm{ratio}=\frac{\mathrm{Absolute}({PC1}_{\mathrm{trisomy}}-{PC1}_{\mathrm{control}})}{\mathrm{Maximum}({PC1}_{\mathrm{trisomy}}\;\&\;{PC1}_{\mathrm{control}})}$$

For altered A/B compartment region definition, the A/B compartment regions with change ratio > 0.5 were considered altered; otherwise, the regions were considered conserved. Additionally, the regions with differential A/B compartment types between samples were considered altered.

### Spatial conformation feature enrichment analysis

Enrichment analysis was performed to determine whether TADs, chromatin loops, and A/B compartments were significantly enriched in abnormal CNV regions, high SNP density regions, and consistent regions. Statistical significance was assessed using Chi-square test. The odds ratio and 95% confidence interval were calculated with the fmsb package in R. The abnormal CNV regions refer to regions with significant CNV events in the trisomy sample but not in the control sample. The high SNP density regions refer to regions with at least twice the number of SNPs in the trisomy sample compared to the control sample and with the number of differential SNPs exceeding 100 per 100,000 bp. The consistent regions were defined as regions containing no significant CNV events and low SNP number (less than 10 SNPs per 100,000 bp).

### Predicting gene expression in trisomy

Gene expression in trisomy was predicted using the gene expression profile of the control sample along with three additional features: copy number value, eQTL fold change, and Hi-C similarity value. The copy number value was derived from significant CNV events, and genes without significant CNV events were assigned a value of 1. The eQTL fold change was obtained from the GTEx portal, with results selected from the brain cortex as the suitable feature. The Hi-C similarity value was calculated based on the gene body region between the trisomy patient and the healthy control. The predicted gene expression value in trisomy was calculated as follows:


(2) $$E_{\mathrm{trisomy}}=E_{\mathrm{control}}\frac{{VC}_{\mathrm{trosomy}}\prod_{i=1}^{n}{VF}_{{\mathrm{trisomy}}_{i}}}{{VC}_{\mathrm{control}}\prod_{j=1}^{m}{VF}_{{\mathrm{control}}_{j}}}V_{\mathrm{similarity}}$$

where *E* stands for gene expression, *VC* stands for copy number value, *VF* stands for eQTL fold change, *n* means the number of eQTLs in the trisomy patient, *m* means the number of eQTLs in the healthy control, and *V_similarity_* stands for Hi-C similarity value between the trisomy patient and the healthy control.

### Integrated haplotype-resolved multi-omics analysis strategy

#### Separation of fragments using parental-specific SNPs

Based on SNP profiles of the parents and the trisomy patient, SNPs were classified into three categories: paternal-specific, maternal-specific, and shared SNPs. Shared SNPs were defined as those present in the genomes of both parents and the trisomy fetus, and specific SNPs were defined as those present in the genomes of one parent and the trisomy fetus. SNPs detected only in the genome of one parent but not in the trisomy fetus were also defined as specific SNPs, while SNPs detected only in the genome of the trisomy fetus were excluded. Sequencing fragments with paternal-specific SNPs were designated as paternal-specific fragments, and those with maternal-specific SNPs were termed maternal-specific fragments.

#### Haplotype-resolved CNV analysis

Haplotype-resolved CNV analysis was performed using WGS fragments assigned to paternal-specific and maternal-specific categories. Fragment frequencies then were quantified in 100-kb genomic bins. These bins were then clustered using a *k*-means algorithm to get the true chromosome-wide copy number distribution for each haplotype.

#### Haplotype-resolved gene expression analysis

Haplotype-resolved gene expression analysis was conducted using RNA-seq fragments assigned to paternal-specific and maternal-specific categories. Read counts per exon were quantified with HTSeq (v0.13.5) [29]. Normalization and differential expression analysis between haplotypes were performed using DESeq2 (v1.36.0) [30], with the read counts on the genes as input.

#### Haplotype-resolved Hi-C analysis

Haplotype-resolved Hi-C strategy was implemented as described below. Following the separation of sequencing reads into paternal-specific and maternal-specific reads, we assigned the interaction events to either a paternal-specific or maternal-specific contact matrix. Each dot in the specific contact matrix means the interaction intensity between two genomic loci.

### SNP density analysis

SNP density was calculated to assess the distribution differences of SNPs between sister chromatids. For each defined region, SNPs were counted in bins of a specified size. The difference in SNP counts between sister chromatids was then calculated for each bin, and the sum of these differences across all bins was defined as SNP density.

### Motif enrichment analysis

Motif scanning was performed using findMotifsGenome.pl from Hypergeometric Optimization of Motif EnRichment (HOMER) software (v4.11) [34] and Find Individual Motif Occurences (FIMO) from Multiple Em for Motif Elicitation (MEME) Suite (v5.4.1) [35] with default parameters.

### Information sourced from public databases

Histone modification and chromatin accessibility data of human fetal samples were obtained from the National Institutes of Health (NIH) Roadmap Epigenomics Project (https://www.ncbi.nlm.nih.gov/geo/roadmap/epigenomics/). DNA methylation data of DS patients were obtained from the Gene Expression Omnibus (GEO: GSE73747). Chromatin state segmentation data of each cell line were obtained from the Encyclopedia of DNA Elements (ENCODE) at UCSC Genome Browser (http://genome.ucsc.edu/).

## Ethical statement

This study was approved by the Medical Ethics Committee of Taihe Hospital, Hubei University of Medicine, Shiyan, China (Approved No. 201928), and complies with all relevant ethical regulations. Written informed consent was provided either directly by adult participants or by the legal guardians of fetuses.

## Code availability

The scripts for omics analysis are available at BioCode at the National Genomics Data Center (NGDC), China National Center for Bioinformation (CNCB) (BioCode: BT007956), which are publicly accessible at https://ngdc.cncb.ac.cn/biocode/tool/BT007956.

## Supplementary Material

qzaf054_Supplementary_Data

## Data Availability

The raw sequence data reported in this study have been deposited in the Genome Sequence Archive for Human [36] at the NGDC, CNCB (GSA-Human: HRA003660), and are publicly accessible at https://ngdc.cncb.ac.cn/gsa-human. The processed data reported in this study have been deposited in the Open Archive for Miscellaneous Data [36] at the NGDC, CNCB (OMIX: OMIX002650 for RNA-seq data, OMIX002651 for Hi-C data, OMIX002652 for CNV data, OMIX005023 for haplotype-resolved RNA-seq data, OMIX005024 for haplotype-resolved Hi-C data, and OMIX005025 for haplotype-resolved CNV data), and are publicly accessible at https://ngdc.cncb.ac.cn/omix.

## CRediT author statement

**Chengchao Wu:** Conceptualization, Methodology, Supervision, Data curation, Formal analysis, Software, Writing – original draft, Visualization, Writing – review & editing, Project administration, Validation. **Tianshu Zhou:** Investigation. **Wenfu Ke:** Investigation. **Wei Xiong:** Investigation, Resources. **Zhihui Zhang:** Investigation. **Siheng Zhang:** Investigation, Resources. **Jinyue Wang:** Investigation. **Lulu Deng:** Data curation, Formal analysis, Software. **Keji Yan:** Investigation. **Man Wang:** Resources. **Shenglong He:** Resources. **Qi Gong:** Resources. **Chao Ma:** Resources. **Xiaping Chen:** Resources. **Yan Li:** Resources. **He Long:** Funding acquisition. **Chong Guo:** Project administration, Validation. **Gang Cao:** Conceptualization, Methodology, Supervision, Writing – original draft, Visualization, Writing – review & editing, Project administration, Validation, Funding acquisition. **Zhijun Zhang:** Project administration, Validation, Funding acquisition, Conceptualization, Methodology, Supervision. All authors have read and approved the final manuscript.

## ORCID

0000-0002-7508-3379 (Chengchao Wu)

0000-0003-3555-0611 (Tianshu Zhou)

0009-0009-8049-8630 (Wenfu Ke)

0009-0007-5788-8564 (Wei Xiong)

0009-0001-4432-386X (Zhihui Zhang)

0009-0002-0441-2689 (Siheng Zhang)

0009-0005-1176-2136 (Jinyue Wang)

0000-0001-6800-4954 (Lulu Deng)

0000-0003-4557-4078 (Keji Yan)

0009-0000-8842-2600 (Man Wang)

0009-0001-5925-4692 (Shenglong He)

0009-0007-6404-7229 (Qi Gong)

0009-0006-6501-2311 (Chao Ma)

0009-0003-2768-502X (Xiaping Chen)

0009-0005-3308-0398 (Yan Li)

0000-0002-8494-8258 (He Long)

0000-0003-3101-8564 (Chong Guo)

0000-0002-3020-5379 (Gang Cao)

0000-0002-1290-2429 (Zhijun Zhang)
