# Supplementary material for: Deciphering Haplotype-level Chromosome Conformation Alteration in Down Syndrome by Haplotype-resolved Multi-omics Analysis
Source: Genomics Proteomics Bioinformatics. 2025 Jun 12;23(4):qzaf054. doi: 10.1093/gpbjnl/qzaf054 (PMC12571509; doi:10.1093/gpbjnl/qzaf054)
Supplement: qzaf054_Supplementary_Data [file qzaf054_supplementary_data.zip › Figure_S4.pdf]

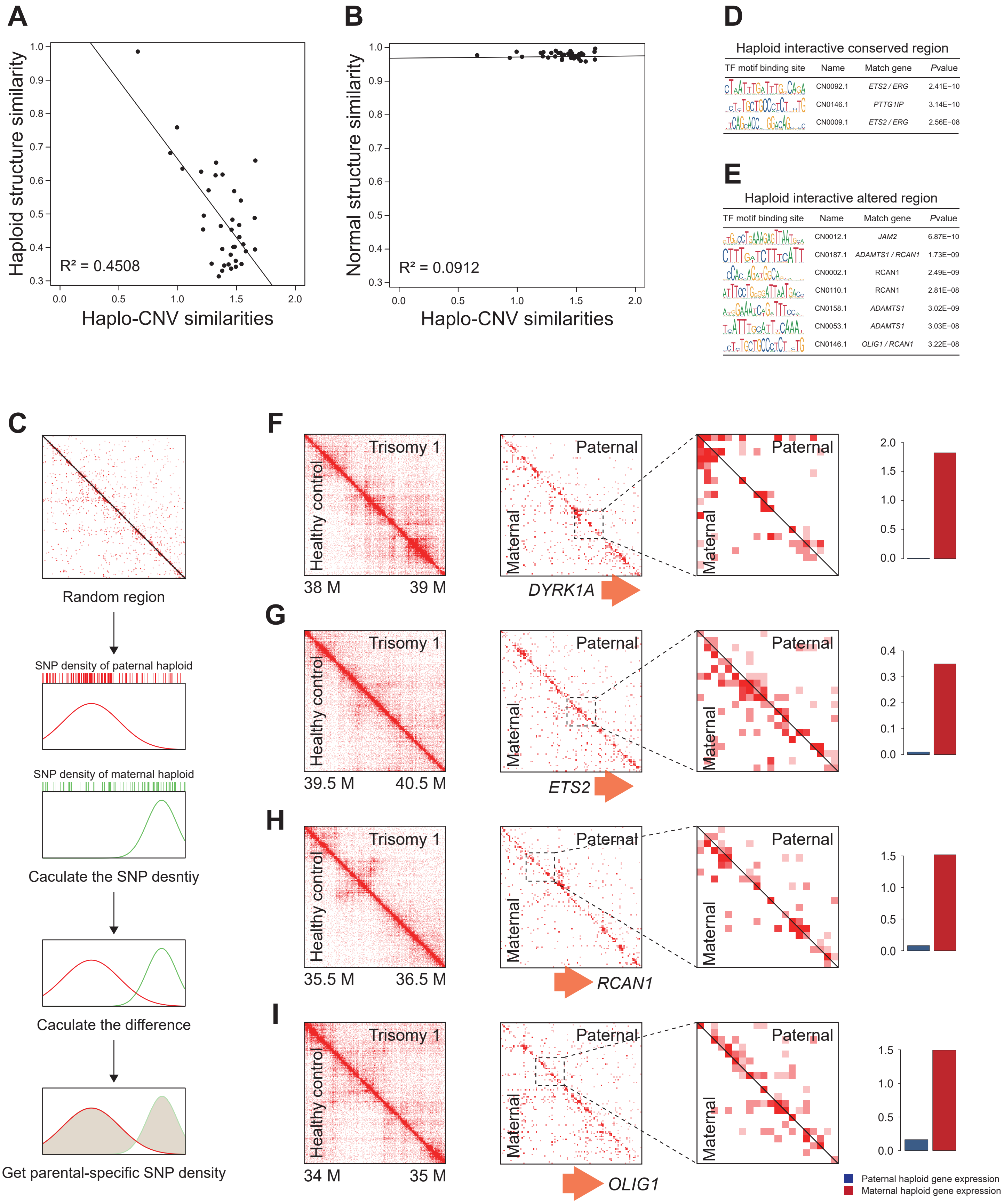

A
Haploid structure similarity
Haplo-CNV similarities
R² = 0.4508
B
Normal structure similarity
Haplo-CNV similarities
R² = 0.0912
D
Haploid interactive conserved region
TF motif binding site | Name | Match gene | Pvalue
CN0092.1 | ETS2 / ERG | 2.41E−10
CN0146.1 | PTTG1IP | 3.14E−10
CN0009.1 | ETS2 / ERG | 2.56E−08
E
Haploid interactive altered region
TF motif binding site | Name | Match gene | Pvalue
CN0012.1 | JAM2 | 6.87E−10
CN0187.1 | ADAMTS1 / RCAN1 | 1.73E−09
CN0002.1 | RCAN1 | 2.49E−09
CN0110.1 | RCAN1 | 2.81E−08
CN0158.1 | ADAMTS1 | 3.02E−09
CN0053.1 | ADAMTS1 | 3.03E−08
CN0146.1 | OLIG1 / RCAN1 | 3.22E−08
C
Random region
SNP density of paternal haploid
SNP density of maternal haploid
Caculate the SNP desntiy
Caculate the difference
Get parental-specific SNP density
F
Trisomy 1
Healthy control
38 M
39 M
Paternal
Maternal
DYRK1A
G
39.5 M
40.5 M
ETS2
H
35.5 M
36.5 M
RCAN1
I
34 M
35 M
OLIG1
Paternal haploid gene expression
Maternal haploid gene expression
