# Supplementary material for: Deciphering Haplotype-level Chromosome Conformation Alteration in Down Syndrome by Haplotype-resolved Multi-omics Analysis
Source: Genomics Proteomics Bioinformatics. 2025 Jun 12;23(4):qzaf054. doi: 10.1093/gpbjnl/qzaf054 (PMC12571509; doi:10.1093/gpbjnl/qzaf054)
Supplement: qzaf054_Supplementary_Data [file qzaf054_supplementary_data.zip › supplementary material captions.docx]

**Supplementary material**

**Figure S1 Detail analysis of SNP densities, CNVs, and gene expressions**

**A.** SNP counts of four samples in whole genome. **B.** Venn plot of SNP counts between trisomy id1 and the parents. **C.**–**E.** Copy number atlas of trisomy parents and healthy control. **F.** Ratio of copy number between trisomy id1 and control id5 in whole genome and HSA21. **G.** Heatmap of significant different expression gene in HSA21. **H.** Differences in present splice inclusion (ΔPSI) of alternative splicing transcripts between trisomy id1 and control id5 in HSA21. DS-related genes which have significant ΔPSI were marked out.

**Figure S2 Hi-C differences between trisomy patient and healthy control**

**A.** Repeatability of interactive matrices in two replication libraries in trisomy id1–4 and control id5. **B.** Interactive matrices correlations in the whole genome level between trisomy id1–4 and control id5. **C.** Spatial chromosome interactive matrices heatmap of trisomy id1–4 and control id5 on HSA21:35,500,000–36,100,000. Black arrowhead points out the two loops existed nearby the gene *RCAN1* in the trisomy id1–4. **D**. Histone modification signals of human brain H3K27me3 and DNA methylation levels in this region. **E.** Gene expression of *RCAN1* in trisomy id1 and control id5. **F.** Inter-chromosomal interactives with HSA21 between trisomy id1–4 and control id5. **G.** Percentage of inter-chromosomal interactive intensity above 100 between trisomy patient and healthy control. **H.** Diagram of predicting gene expression in trisomy with one-dimensional and three-dimensional features. **I.** Hi-C similarities between trisomy id1 and control id5 in true corrected gene and false corrected gene region. TSS, Transcription Start Site;

**Figure S3 Haploid Hi-C and related analysis**

**A.** and **B.** Percentage of haploid copy number of each chromosome in paternal haploid (A) and maternal haploid (B). **C.** CNV of trisomy patient and corresponding haploid CNV in HSA21. **D.** Copy number proportion of long-arm and short-arm in HSA21. **E.** Pets number of paternal and maternal haploid Hi-C in each chromosome. **F.** Paternal and maternal pets’ number of inter-chromosome, intra-chromosome, and inter-haploid. **G.** Interactive intensities between paternal and maternal haploids with different bin sizes.

**Figure S4 Correlation between different multiple haploid omics analysis**

**A.** Linear correlation of differential haploid CNVs in each haploid Hi-C similarity levels. **B.** Linear correlation of differential haploid CNVs in each Hi-C similarity levels. **C.** Diagram of calculate the SNP density in paternal and maternal haploids. **D.** and **E.** TF binding site in conserved and altered region of haploid interactive between paternal and maternal haploids. **F.**–**I.** Hi-C interactive matrices and haploid Hi-C interactive matrices in the neighboring regions of four genes. Haploid gene expression was shown in the bar plot. TF, transcription factor.

**Table S1 The detailed information of DEGs between trisomy id1 and control id5**

**Table S2 The QC matrices of general DLO Hi-C datasets and haploid Hi-C datasets**

**Table S3 The detailed information of TADs, loops and A/B compartments**

**Table S4 The detailed information of DEGs predicted with one-dimensional genome features**
