# Supplementary figures and images for: Deciphering Haplotype-level Chromosome Conformation Alteration in Down Syndrome by Haplotype-resolved Multi-omics Analysis
Source: Genomics Proteomics Bioinformatics. 2025 Jun 12;23(4):qzaf054. doi: 10.1093/gpbjnl/qzaf054 (PMC12571509; doi:10.1093/gpbjnl/qzaf054)

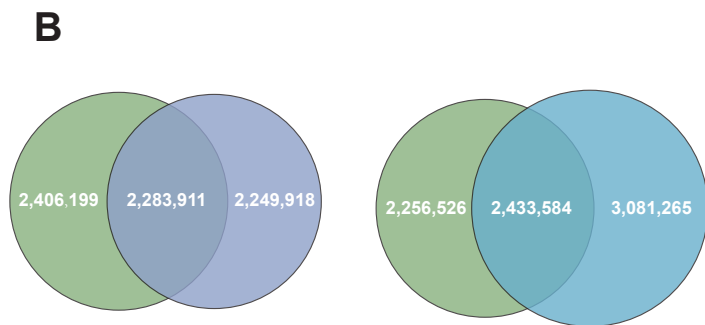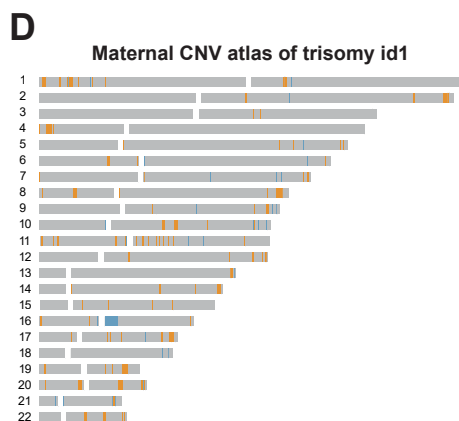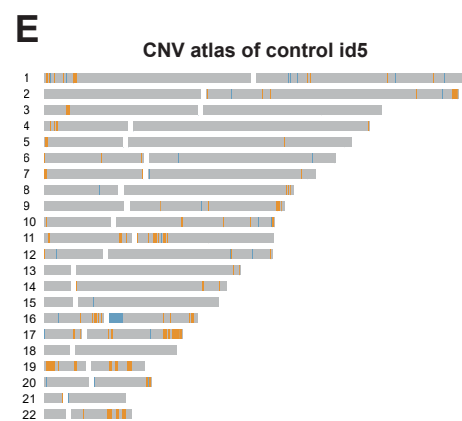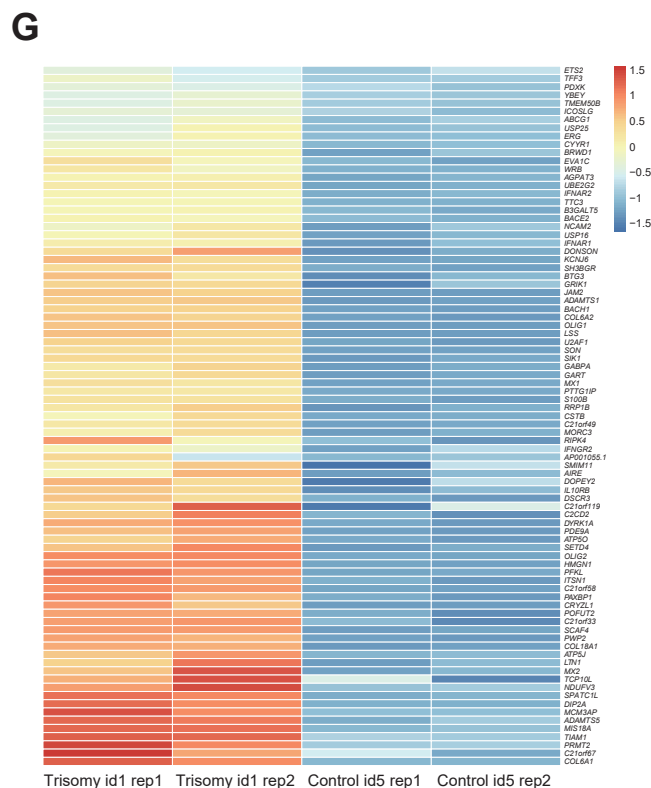

Supplement: qzaf054_Supplementary_Data [file qzaf054_supplementary_data.zip › Figure_S1.pdf]

**A**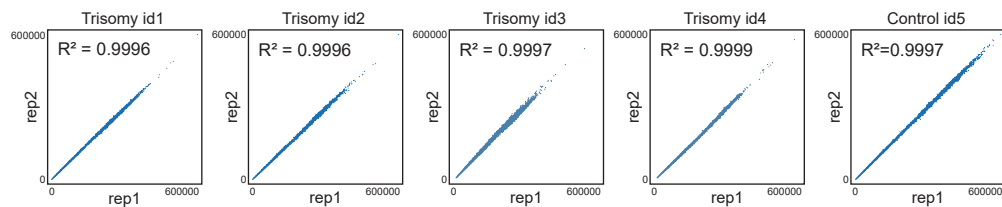**B**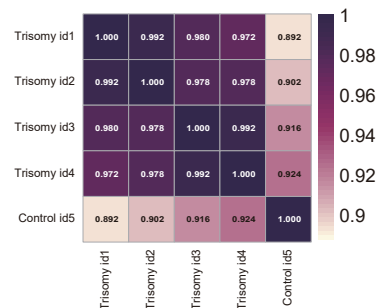**C**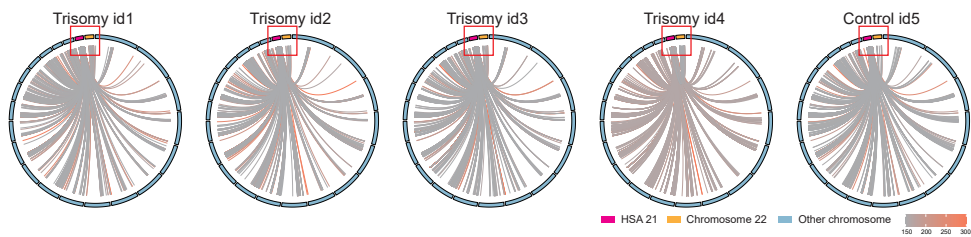**D**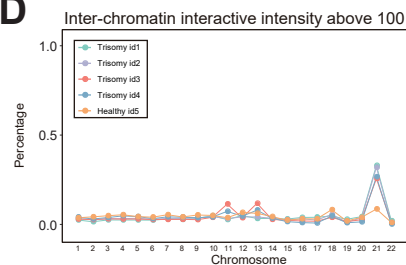**E**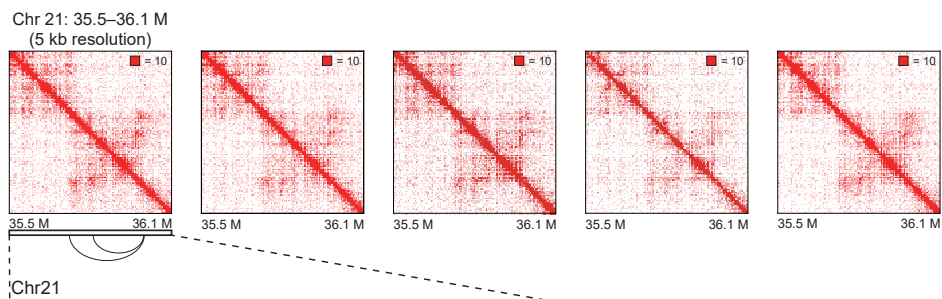**F**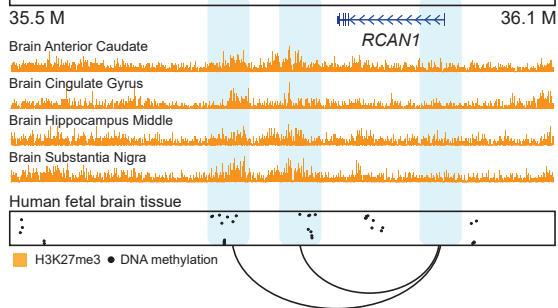**H**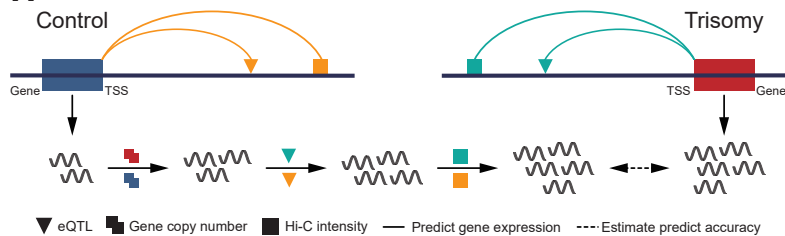**G**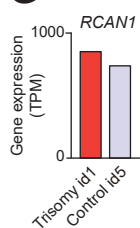**I**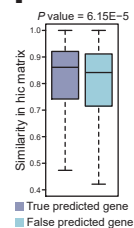

Supplement: qzaf054_Supplementary_Data [file qzaf054_supplementary_data.zip › Figure_S2.pdf]

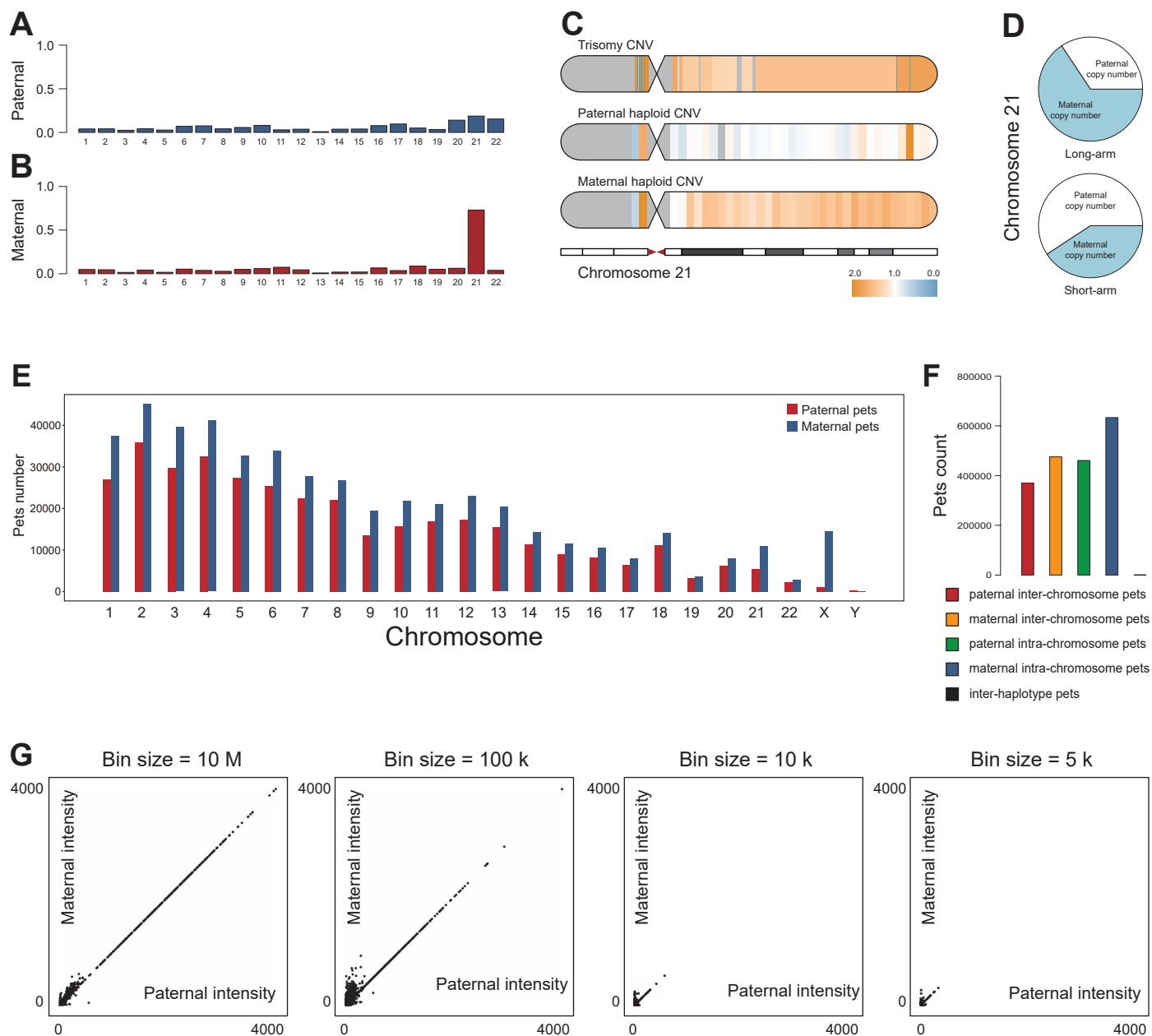

Supplement: qzaf054_Supplementary_Data [file qzaf054_supplementary_data.zip › Figure_S3.pdf]
